# Vascular tortuosity in relationship with hypertension and posterior fossa volume in hemifacial spasm

**DOI:** 10.1186/s12883-016-0634-z

**Published:** 2016-07-29

**Authors:** Evan Cyril Edmond, Samantha Xue-Li Sim, Hui-Hua Li, Eng-King Tan, Ling-Ling Chan

**Affiliations:** 1 Department of Diagnostic Radiology, Singapore General Hospital, Singapore, 169608 Singapore; 2 Health Services Research and Biostatistics Unit, Division of Research, Singapore General Hospital, Singapore, 169608 Singapore; 3 Department of Neurology, National Neuroscience Institute, Singapore General Hospital, Singapore, 169608 Singapore; 4 Duke NUS Graduate Medical School, Singapore, 169857 Singapore; 5 Oxford Medical School, Oxford, UK

**Keywords:** Hemifacial spasm, Tortuosity, Imaging

## Abstract

**Background:**

Hemifacial spasm (HFS) is a disabling neurological condition. Vascular tortuosity in HFS patients has not been quantified objectively and its relationship with hypertension and posterior fossa volume (PF) is unknown. In a case control magnetic resonance imaging and angiographic (MRI/A) study, we quantified and compared the vascular tortuosity in HFS and controls, and evaluated its relationship with hypertension and PF.

**Methods:**

Using a commercially available vessel probe tool, an index of tortuosity based on vessel over chord length was employed to quantify vascular tortuosity of the vertebral (VA) and basilar arteries (BA) in 79 subjects (40 HFS, 39 controls).

**Results:**

The tortuosity index of the BA (1.09 vs 1.16, *p* = 0.26, 95 % CI 1.07, 1.23), RVA (1.15 vs 1.15, *p* = 0.83, 95 % CI 1.06, 1.38) and LVA (1.14 vs 1.288, *p* = 0.16, 95 % CI 1.14, 1.44) was not different between HFS and controls, with adjustments for PF volume and hypertension.

**Conclusions:**

Contrary to popular belief, our study showed that taking into account hypertension and PF volume, vascular tortuosity of the vertebrobasilar arteries is unlikely to be a major etiologic factor in HFS, though its role in select individuals cannot be excluded. The complex interplay of facial nerve hyperexcitability, genetic predisposition, vascular tortuosity, posterior fossa volume and hypertension needs to be further evaluated.

## Background

Hemifacial spasm (HFS) is characterized by intermittent, unilateral, and involuntary contractions of the facial muscles and neurovascular conflict at the facial root exit zone (REZ), and is widely recognized as the most likely underlying etiology [1–13]. The facial REZ from the brainstem is a segment of about 10 mm lying between central and peripheral myelination of axons. This reduced myelination predisposes to nerve dysfunction if lesions affect the nerve at this anatomical site. Pulsatile vascular compression of the facial nerve at this site has been implicated as the cause [8, 13]. The culprit vessels are generally loops of the anterior inferior cerebellar artery, posterior inferior cerebellar artery or vertebral artery. As a result, surgical microvascular decompression to relieve the nerve root has become an established treatment of this condition [8, 13].

A number of potential predisposing factors to the development of HFS have been investigated, including female sex, increased vascular tortuosity [8], reduced posterior fossa volume [4, 6], and arterial hypertension [3, 4, 11, 12].

Vascular tortuosity is a subjective concept, and descriptions of tortuosity in HFS have included REZ compression caused by dolichoectasia, localised vascular tortuosity, or more generalised tortuosity of the vertebrobasilar system. Objective approaches to automated analysis of tortuosity have been varied, with the most applications in the analysis of retinal vascular tortuosity with two dimensional images. Analysis of tortuosity in three-dimensional images of cerebral vasculature is usually based on subjective qualitative assessments. To our knowledge, there have been no published data on quantitative evaluation of vascular tortuosity in HFS.

To address the current gaps in knowledge, we studied the tortuosity index in a case control cohort of HFS. Using magnetic resonance angiography (MRA), we calculated the vertebrobasilar tortuosity index and examined its association with HFS, and its interaction with factors such as posterior fossa CSF volume and arterial hypertension.

## Methods

### Clinical

HFS patients were diagnosed by a movement disorders neurologist and consented to MRI/MRA evaluation to exclude structural and vascular abnormalities in the posterior fossa as part of clinical care. These patients gave verbal informed consent for the MRI/A and their data anonymised for the analysis. A history of hypertension was recorded. Controls were volunteers without HFS, matched one-to-one for age, gender and hypertension and gave written informed consent for the imaging research study. This study was approved by the Singapore General Hospital ethics committee.

### Imaging

All subjects underwent a standardized MRI protocol as previously described [3, 4, 10, 14]. This included the 3-dimensional constructive interference at steady state (CISS) sequence focused over the posterior fossa (repetition time [TR] 12, echo time [TE] 6, flip angle [FA] 70, number of excitations [NEX] 2, 282 ϫ 512 matrix, 0.75-mm partitions, 56 slices) and time-of-flight MRA (TR 35, TE 7.2, FA 20, NEX 1, 210 ϫ 512 matrix, 0.8-mm partitions, 96 slices) sequence. A screening fluid-attenuated inversion recovery (TR 9,000 msec, TE 110 msec, inversion recovery 2,500 msec, NEX 1) scan of the brain was also obtained, which excluded the presence of brainstem and bony abnormalities, or space-occupying lesions. All the axial sequences were acquired parallel to the bicommissural line.

### Image analysis

**Fig. 1:**
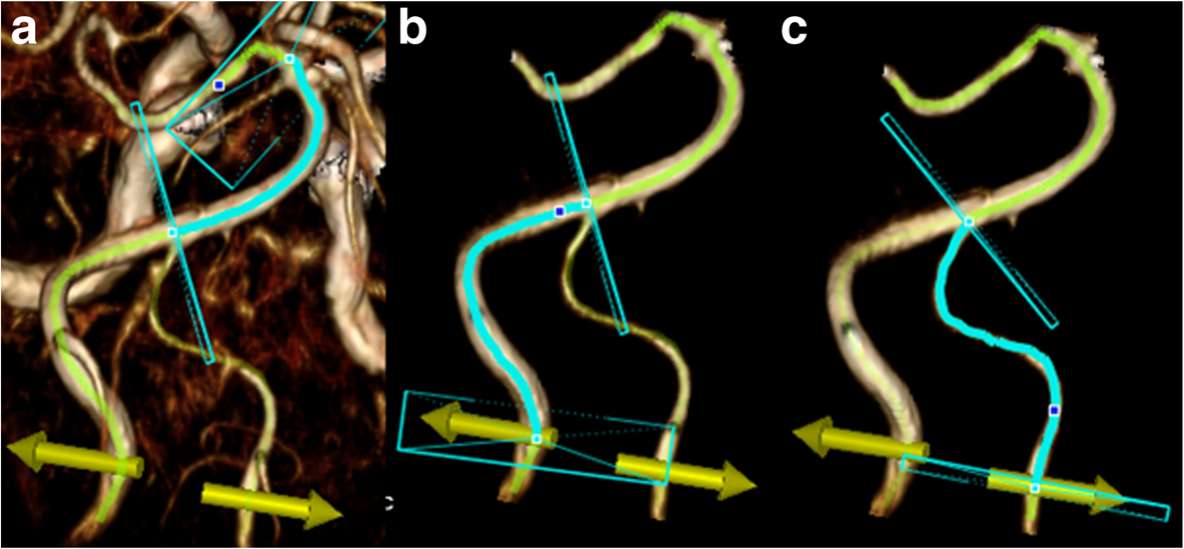
Three-dimensional illustration of vessel selection (*blue* square dot), centreline extraction (curvilinear *yellow* line) and length measurements (curvilinear blue line) of the **a** basilar artery, **b** dominant left vertebral and **c** hypoplastic right vertebral arteries, a view from behind. Yellow arrows indicate the horizontal level of the hypoglossal canals. The internal carotid arteries in the background in **a** are masked in **b** and **c**. The vessel length (Lv) is defined as the length of the centreline extraction of each arterial segment and the chord length (Lc) as the shortest distance between the origin and termination point of each arterial segment. The Lv and Lc were measured for each of the three arterial segments, and the tortuosity index derived as the ratio, Lv/Lc

Semi-automated measurement of tortuosity was performed using the Toshiba VITAL Images’ Advanced Visualization application. The Vessel Probe tool allows automated vessel selection and centreline extraction of the vertebrobasilar arteries on the MRA sequence (Fig 1). Two trained raters independently defined segments of the vessel as the left vertebral artery (LVA), right vertebral artery (RVA) and basilar artery (BA). The origin of each vertebral artery was defined as the entry point into the axial plane containing the hypoglossal canals, and the terminations as the confluence point at the origin of the basilar artery. The termination of the basilar artery is defined at the point where it bifurcates into the posterior cerebral arteries. The vessel length (Lv) is defined as the length of the centreline extraction of each arterial segment and the chord length (Lc) as the shortest distance between the origin and termination point of each arterial segment. The Lv and Lc were measured for each of the three arterial segments, and the tortuosity index derived as the ratio, Lv/Lc. The volume of the posterior fossa cerebrospinal fluid (CSF) space was also quantified using previous methodology [4] on the CISS sequence, centred over the internal auditory canals.

### Statistical methods

Median, together with interquartile range (IQR), was reported for continuous variables, while frequency, together with proportion, was reported for categorical data. The Mann-Whitney *U* test was carried out to compare the difference of continuous variables between different groups of patients, while Fisher’s exact test was performed to compare the distribution of categorical data between different groups of patients. All analyses were done using R 3.1.3 (https://www.r-project.org/). A logistic regression analysis with age, gender, posterior fossa volume and hypertension as independent variables and the tortuosity index in the BA, RVA and LVA in HFS and controls as dependent variables were also carried out. Statistical significance was defined at *p* < 0.05.

The inter rater reliability of the tortuosity index was calculated using the intraclass correlation (ICC) which is a useful estimate of inter-rater reliability on quantitative data. A value between 0–0.2 indicates *poor* agreement, 0.3–0.4 indicates *fair* agreement, 0.5–0.6 indicates moderate agreement, 0.7–0.8 indicates *strong* agreement; and >0.8 indicates *almost perfect* agreement.

## Results

A total of 79 study subjects (40 HFS and 39 controls) were included. Table 1 listed the characteristics of HFS patients and controls. There was no statistically significant difference in age and gender. The ICC for the tortuosity index between the two raters was near perfect. The ICC for BA tortuosity was 0.922 (95 % CI 0.881, 0.949), RVA tortuosity 0.971 (95 % CI 0.955, 0.981) and LVA tortuosity 0.978 (95 % CI 0.965, 0.986).

**Table 1:** Comparison of demographic and vascular characteristics between HFS patients and controls

	HFS (n-40)	HC (*n* = 39)	*P*-value
Age (Median IQR)	53.5 (48.8, 58.3)	56.0 (50.0, 58.5)	0.5824
Sex			1.0000
Female	25 (62.5 %)	25 (64.1 %)	
Male	15 (37.5 %)	14 (35.9 %)	
Hypertension			1.0000
No	18 (45.0 %)	18 (46.2 %)	
Yes	22 (55.0 %)	21 (53.8 %)	
BA tortuosity (Median IQR)	1.115 (1.065, 1.225)	1.120 (1.073, 1.172)	0.9843
RVA tortuosity (Median IQR)	1.150 (1.074, 1.288)	1.150 (1.090, 1.278)	0.6624
LVA tortuosity (Median IQR)	1.200 (1.062, 1.397)	1.190 (1.078, 1.368)	0.9961

The VA was congenitally absent or hypoplastic and could not be detected by the software in 3 patients (1 right, 2 left) and 5 controls (2 right, 3 left) respectively. The tortuosity index of the BA (1.09 vs 1.16, *p* = 0.26, 95 % CI 1.07, 1.23), RVA (1.15 vs 1.15, *p* = 0.83, 95 % CI 1.06, 1.38) and LVA (1.14 vs 1.288, *p* = 0.16, 95 % CI 1.14, 1.44) was not different between HFS and controls, with or without adjustments for age, gender, posterior fossa volume and hypertension (Tables 1 and 2). Linear regression analysis adjusted by, duration of HFS, hypertension and posterior fossa CSF volume among patients with HFS also did not reveal any significant correlation between vascular tortuosity index of the vertebrobasilar arteries with HFS (Table 3). The tortuosity index between right and left side was also not different in those with different VA dominance (Table 4).

**Table 2:** Linear regression adjusted by hypertension, posterior fossa volume, gender and age among all subjects

	HFS-control	*P*-value
BA tortuosity (Median IQR)	0.010 (–0.040, 0.060)	0.6980
RVA tortuosity (Median IQR)	−0.012 (–0.094, 0.070)	0.7820
LVA tortuosity (Median IQR)	0.019 (–0.087, 0.124)	0.7320

**Table 3:** Linear regression adjusted by duration of HFS, hypertension and posterior fossa volume among patients with HFS only

	Right-Left	*P*-value
BA tortuosity (Median IQR)	−0.072 (–0.153, 0.009)	0.090
RVA tortuosity (Median IQR)	0.065 (–0.054, 0.183)	0.290
LVA tortuosity (Median IQR)	−0.130 (–0.295, 0.035)	0.130

**Table 4:** Linear regression adjusted by duration of HFS, hypertension and posterior fossa volume between patients with different VA dominance

	Right-Left	*P*-value
BA tortuosity (Median IQR)	−0.080 (–0.265, 0.106)	0.410
RVA tortuosity (Median IQR)	0.148 (–0.060, 0.356)	0.179
LVA tortuosity (Median IQR)	−0.131 (–0.492, 0.230)	0.486

## Discussion

HFS is a disabling neurological disorder and can significantly affect quality of life [14, 15]. Neurovascular conflict has been widely recognized as the most common underlying etiology of HFS [2, 3, 16]. Vascular tortuosity has also been frequently linked as a predisposing factor for the neurovascular conflict [8]. While observations of vascular tortuosity have been reported during surgical decompression surgery for HFS [8], such assessments are usually qualitative and may be subjected to observer bias and limited surgical window. To our knowledge, case control quantitative assessment of vascular tortuosity in relationship with hypertension and posterior fossa volume has not been examined in HFS.

Tortuosity is a subjective concept with a variety of mathematical models ranging from more simple to complex [17]. Whilst complex mathematical models that may more closely approximate observer rated tortuosity have been demonstrated in MR angiography of intracerebral vasculature [18], even very simple indices can account for the majority of the variation in observer rated tortuosity. As such, a simple index was chosen for this study, comparing the luminal vascular course to the shortest distance between its origin and endpoint.

Here we demonstrated the utility of a simple tortuosity index in 3 dimensions involving the vertebrobasilar artery system on MR angiography using a case control methodology. Since age, gender and arterial hypertension may be predisposing factors in HFS, we have recruited controls of similar age, gender and with similar frequency of hypertension as HFS. The inter-rater reliability of our assessment of the tortuosity index was assessed using the ICC. The very high ICC values (0.95 to 0.98) for the 3 vessels we examined indicated near perfect agreement between our two raters, supporting the reliability of the measurements based on reproducible and comparable landmarks.

Interestingly, our study demonstrated that the tortuosity index in the BA, RVA and LVA was not different between HFS and controls and this was independent of age, gender, hypertension and posterior fossa volume. In the analysis of tortuosity in hypertension (both cases and controls), the largest increase of tortuosity in hypertension was seen in the left vertebral artery. The presence of a dominant vertebral artery and angulation of the vertebrobasilar junction has been suggested to be linked to HFS [8].

While the negative association of objective vascular tortuosity of the BA, RVA and LVA with HFS may be surprising, our findings support what is believed to be a complex interplay of multiple factors in the pathogenesis of HFS. It is likely that reduced posterior fossa CSF volume, increased vascular tortuosity, or hypertension alone may not be the single or most important overriding predisposing factor. Individual susceptibility of the facial motor nucleus hyperactivity and its compensation potential or genetics [19] may also explain why only certain at-risk individuals develop HFS while others with similar or more risk factors do not. Our study has some limitations. We were only able to quantify the tortuosity index of the BA, RVA and LVA. The current study was not designed to evaluate the tortuosity index of the smaller arteries such as the anterior inferior cerebellar artery (AICA) and posterior inferior cerebellar artery (PICA). Based on the image sequences we have acquired, this could not be meaningfully carried out. Importantly, there is a lack of standardized end points for these vessels and hence it is difficult to evaluate. We think that vascular tortuosity in the bigger vessels is more relevant in association studies involving the size of the posterior fossa volume and this is the focus of our study. In addition, we believe the contact between the REZ and the vessel is really more important than vascular tortuosity, even though the latter may play a role.

## Conclusions

We demonstrated for the first time that tortuosity of the vertebrobasilar arteries can be objectively and simply quantified in the posterior fossa in HFS. Contrary to popular belief, our results suggest that taking into account hypertension and posterior fossa volume, vascular tortuosity alone is not a major factor in the etiology of HFS, though its role in select individuals cannot be totally excluded. The complex interplay of facial nerve hyperexcitability, genetic predisposition, vascular tortuosity, posterior fossa volume and hypertension needs to be further evaluated.

## Abbreviations

AICA, anterior inferior cerebellar artery; BA, basilar arteries; CISS, constructive interference at steady state; CSF, cerebrospinal fluid; FA, flip angle; HFS, hemifacial spasm; ICC, intraclass correlation; IQR, interquartile range; Lc, chord length; Lv, vessel length; MRA, magnetic resonance angiography; MRI, magnetic resonance imaging and angiographic; NEX, number of excitations; PF, posterior fossa volume; PICA, posterior inferior cerebellar artery; REZ, root exit zone; TE, echo time; TR, repetition time; VA, vertebral artery

## References

[1] Wang A, Jankovic J (1998). Hemifacial spasm: clinical findings and treatment. Muscle Nerve.

[2] Jannetta PJ, Kassam A (1999). Hemifacial spasm. J Neurol Neurosurg Psychiatry.

[3] Chan LL, Lo YL, Lee E, Fook-Chong S, Tan EK (2005). Ventrolateral medullary compression in hypertensive patients with hemifacial spasm. Neurology.

[4] Chan LL, Ng KM (2009). Three-dimensional MR volumetric analysis of the posterior fossa CSF space in hemifacial spasm. Neurology.

[5] Oliveira LD, Cardoso F, Vargas AP (1999). Hemifacial spasm and arterial hypertension. Mov Disord.

[6] Kamiguchi H, Ohira T, Ochiai M, Kawase T (1997). Computed tomographic analysis of hemifacial spasm: narrowing of the posterior fossa as a possible facilitating factor for neurovascular compression. J Neurol Neurosurg Psychiatry.

[7] Chaudhry N, Srivastava A, Joshi L (2015). Hemifacial spasm: The past, present and future. J Neurol Sci.

[8] Park JS, Koh EJ, Choi HY, Lee JM (2015). Characteristic anatomical conformation of the vertebral artery causing vascular compression against the root exit zone of the facial nerve in patients with hemifacial spasm. Acta Neurochir (Wien).

[9] Sandell T, Holmen J, Eide PK (2013). Hypertension in patients with cranial nerve vascular compression syndromes and comparison with a population-based cohort. J Neurosurg.

[10] Tan EK, Chan LL, Lum SY (2003). Lack of association of Hypertension with Hemifacial Spasm: a clinical and MRI study. Neurology.

[11] Defazio G, Berardelli A, Abbruzzese G (2000). Primary hemifacial spasm and arterial hypertension: a multicenter case-control study. Neurology.

[12] Boogaarts HD, Menovsky T, De Vries J (2012). Primary hypertension and neurovascular compression: a meta-analysis of magnetic resonance imaging studies. J Neurosurg.

[13] Ma Q, Zhang W, Li G (2014). Analysis of therapeutic effect of microvascular decompression surgery on idiopathic hemifacial spasm. J Craniofac Surg.

[14] Tan EK, Fook-Chong S, Lum SY, Thumboo J (2005). Validation of a short disease specific quality of life scale for hemifacial sapsm: correlation with SF 36. J Neurol Neurosurg Psychiatry.

[15] Tan EK, Lum SY, Fook-Chong S, Chan LL, Gabriel C, Lim L (2005). Behind the facial twitch: depressive symptoms in hemifacial spasm. Parkinsonism Relat Disord.

[16] Chan LL, Lee E, Fook-Chong S, Tan EK (2008). Case control MR-CISS and 3D TOF and MRA imaging study of medullary compression in Hemifacial spasm. Mov Disord.

[17] Hart WE, Goldbaum M, Côté B, Kube P, Nelson MR (1999). Measurement and classification of retinal vascular tortuosity. Int J Med Inform.

[18] Bullitt E, Gerig G, Pizer SM, Weili L, Aylward SR (2003). “Measuring tortuosity of the intracerebral vasculature from MRA images,” in Medical Imaging. IEEE Transactions.

[19] Miwa H, Mizuno Y, Kondo T (2002). Familial hemifacial spasm: report of case and review of literature. J Neurol Sci.

